# An Enhanced LoRaWAN Security Protocol for Privacy Preservation in IoT with a Case Study on a Smart Factory-Enabled Parking System

**DOI:** 10.3390/s18061888

**Published:** 2018-06-08

**Authors:** Ilsun You, Soonhyun Kwon, Gaurav Choudhary, Vishal Sharma, Jung Taek Seo

**Affiliations:** Department of Information Security Engineering, Soonchunhyang University, Asan 31538, Korea; ilsunu@gmail.com (I.Y.); tnsgus08@gmail.com (S.K.); gauravchoudhary7777@gmail.com (G.C.); seojt@sch.ac.kr (J.T.S.)

**Keywords:** LoRaWAN, privacy, IoT, smart parking, security, protocol

## Abstract

The Internet of Things (IoT) utilizes algorithms to facilitate intelligent applications across cities in the form of smart-urban projects. As the majority of devices in IoT are battery operated, their applications should be facilitated with a low-power communication setup. Such facility is possible through the Low-Power Wide-Area Network (LPWAN), but at a constrained bit rate. For long-range communication over LPWAN, several approaches and protocols are adopted. One such protocol is the Long-Range Wide Area Network (LoRaWAN), which is a media access layer protocol for long-range communication between the devices and the application servers via LPWAN gateways. However, LoRaWAN comes with fewer security features as a much-secured protocol consumes more battery because of the exorbitant computational overheads. The standard protocol fails to support end-to-end security and perfect forward secrecy while being vulnerable to the replay attack that makes LoRaWAN limited in supporting applications where security (especially end-to-end security) is important. Motivated by this, an enhanced LoRaWAN security protocol is proposed, which not only provides the basic functions of connectivity between the application server and the end device, but additionally averts these listed security issues. The proposed protocol is developed with two options, the Default Option (DO) and the Security-Enhanced Option (SEO). The protocol is validated through Burrows–Abadi–Needham (BAN) logic and the Automated Validation of Internet Security Protocols and Applications (AVISPA) tool. The proposed protocol is also analyzed for overheads through system-based and low-power device-based evaluations. Further, a case study on a smart factory-enabled parking system is considered for its practical application. The results, in terms of network latency with reliability fitting and signaling overheads, show paramount improvements and better performance for the proposed protocol compared with the two handshake options, Pre-Shared Key (PSK) and Elliptic Curve Cryptography (ECC), of Datagram Transport Layer Security (DTLS).

## 1. Introduction

The Internet of Things (IoT) with its principles of “connectivity to all” and “connectivity with all” has become a crucial part of the telecommunication system [1,2,3]. IoT facilities many social issue-solving applications such as smart cities, intelligent transportation, urban surveillance, day-work management and smart-farming [4,5,6,7,8]. With the increasing popularity of IoT, the number of connected devices is bound to increase exponentially. The IoT network can be scaled for improved performance by the use of software-defined and application-aware networking [9,10]. The software technologies can be modeled to enhance the privacy rules and help to enhance the authentication procedures [11,12,13]. With effective strategies for collaborative applications, IoT can be used for long, as well as short distance communications [14,15,16,17].

In the past, IoT was emphasized to incorporate many short-range communications technologies [18,19,20,21,22], which sometimes present an obstacle for efficient working of devices.

As an alternative, Low Power Wide Area Network (LPWAN) technologies are adopted with the property of long-range and low-power computation, but for low-bit rate requirements [23,24,25,26]. However, with the short-distance communications, incorporation of IoT into cyber-physical systems and cloud architectures is always a challenge because of security concerns and a lack of privacy preservation over limited range spectrums [27,28,29]. These security issues worsen when the underlying architecture is used for real-time applications with the involvement of crucial user data [30,31,32,33,34].

Recently, the Long-Range Wide Area Network (LoRaWAN) has become one of the most significant technologies for LPWAN due to its property of long-range communication with energy-efficient computations [35]. These features help to maintain the trade-off between the network latency and the battery lifetime. LoRaWAN also effectively complies with specific features of IoT through the dedicated physical and Medium Access Control (MAC) layer.

The innovative features of the LoRaWAN network are the reasons for compatibility with many low-power applications involving IoT, smart cities, and industrial applications [36,37]. Its advantages are formulated in terms of bandwidth, battery life, range, latency and throughput. Recently, LoRaWAN has been influenced by the standard properties and adopted as a standard mechanism for resource-constrained networks [38].

With the advantages and an enhanced scope of improvements, the LoRaWAN network has already become an emerging area of research. In spite of being well designed, the LoRaWAN network faces several security vulnerabilities, which have been pointed out by many researchers [39,40,41,42]. In more detail, it fulfills the basic security properties, but suffers from the following vulnerabilities. First, its join procedure causes a vulnerability, which leads to exploitation by replay attacks. Second, the protocol cannot provide end-to-end security because the application session key between each device and its application server is established with the help of the core network. In other words, the traffic between the two parties can be easily known by the LoRaWAN network server. Third, the network and application session keys, which are established based on a long-term shared key, cannot provide perfect forward secrecy. Considering that every device can be easily broken and compromised, their long-term key can also be exposed, thereby causing the past session keys and their encrypted data to be recovered. It is obvious that the security flaws mentioned above present an obstacle to the successful settlement of the LoRaWAN network.

In order to address these security flaws, several types of research have been conducted [41,42,43,44]. However, they are lacking in terms of implementation, while maintaining the standard spectrum. Most of them need changes in the existing standard protocol. Therefore, a more secure and effective low-power consumption scheme is required, which is acceptable under the benchmarks of the existing standard. On the other hand, in the LoRaWAN network, it can be considered to apply Datagram Transport Layer Security (DTLS) [45] to provide the end-to-end security between each device and its application server. However, the DTLS handshake procedure results in excessive message signaling and computation overheads, which are not clearly suited for the LoRaWAN network. As an alternative, we can design a lightweight version for the authentication and key exchange between the two parties, which is the strong motivation of this paper.

**Figure 1:**
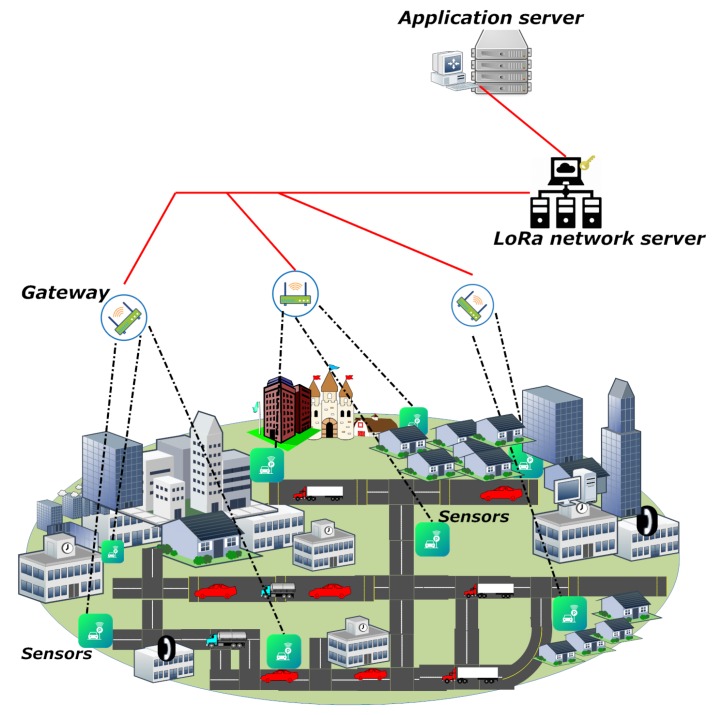
An exemplary illustration of the LoRaWAN-enabled network architecture.

The goal of this paper is to bring out a comprehensive analysis of the LoRaWAN’s security scheme and the existing solutions for its limitations, as well as provide an effective remedy for their problems. Along with such analyses, a secure scheme is proposed to focus on addressing the replay attacks and achieving both the perfect forward secrecy and the end-to-end security between each device and its application server. Note that the proposed protocol can be divided into two parts where the first one is the standard join procedure and the second one is the key exchange protocol. After the proposed protocol (i.e., the strong master session key is established between a device and its application server), the two parties can run the DTLS record protocol based on the established key. The proposed protocol supports the majority of the security properties including mutual authentication, secret key exchange, perfect forward secrecy, end-to-end security and defense against the replay attack to support the security-sensitive applications the should keep the end-to-end security. The proposed protocol is formally analyzed for its security through Burrows–Abadi–Needham (BAN) logic [46] and the Automated Validation of Internet Security Protocols and Applications (AVSIPA) tool [47]. Further, the performance analysis is presented in comparison with the DTLS’s two handshake options, Pre-Shared Key (PSK) and Elliptic Curve Cryptography (ECC), along with a case study on a smart factory-enabled parking system [45,48]. In the case study, the proposed protocol is analyzed for its performance by securing communication between the end devices (sensors) at the parking lot and the application server, which is hosted by the smart factory, as shown in Figure 1. The results are analyzed for network latency with reliability fitting and signaling overheads for the proposed protocol.

The rest of this paper is structured as follows: Section 2 provides details of LoRaWAN and its functionalities. Section 3 presents insight into the related works on LoRaWAN security. Section 4 gives the details on the proposed protocol, its functioning and policies. Analyses through BAN logic and the AVISPA tool are presented in Section 5. Performance evaluations with the smart parking case study are presented in Section 6. Finally, Section 7 concludes the paper along with future directions and remarks.

## 2. Background

This section presents details on LoRaWAN, its architecture, key exchange policies and procedures [49,50]. The basic notations used to describe the LoRaWAN join procedure and the proposed protocol are provided in Table 1.

### 2.1. LoRaWAN Network Architecture

LoRaWAN is designed to be used for battery drain applications where low power consumption with long-range communication is a primary objective. In the LoRaWAN specification v1.02 [51], network range is defined to be 5–15 km, data rates are between 0.3 kbps and 50 kbps and the network is operated over the 868-MHz and 900-MHz ISM bands. LoRaWAN, based on the star topology, has grown as one of the most popular technologies for IoT. Its architecture aims to provide interoperability among IoT devices irrespective of their characteristics.

Its network architecture consists of four entities: device (sensors), gateway, network server and application server. As illustrated in Figure 1, each device is connected to its network server via the corresponding gateway(s) where the device-gateway path is over a single wireless hop and the gateway-network server is interconnected with the non-LoRaWAN network (IP connections). Like the gateway-network server path, the network server communicates with the application servers via a non-LoRaWAN network (IP connections).

### 2.2. Standard LoRaWAN Protocol

In the LoRaWAN network, each device needs to perform a join procedure to enter into the network. The join procedures are classified as Over-The-Air Activation (OTAA) and Activation By Personalization (ABP).

#### 2.2.1. Over-the-Air Activation and Activation by Personalization

In the OTAA mode, a device and its network server mutually authenticate each other and exchange the network and application session keys, *NwkSKey* and *AppSKey*, through the Join procedure. Among the exchanged session keys, the application key *AppSKey* is forwarded to the corresponding application server so that the device and the application server securely exchange data. On the other hand, in ABP mode, it is assumed that the two session keys, *NwkSKey* and *AppSKey*, are stored on their device with the device address *DevAddr*. Therefore, each device can immediately start to communicate with its application server via the LoRaWAN network while skipping the join procedure. This paper focuses on OTAA mode.

#### 2.2.2. Join Procedure

**Figure 2:**
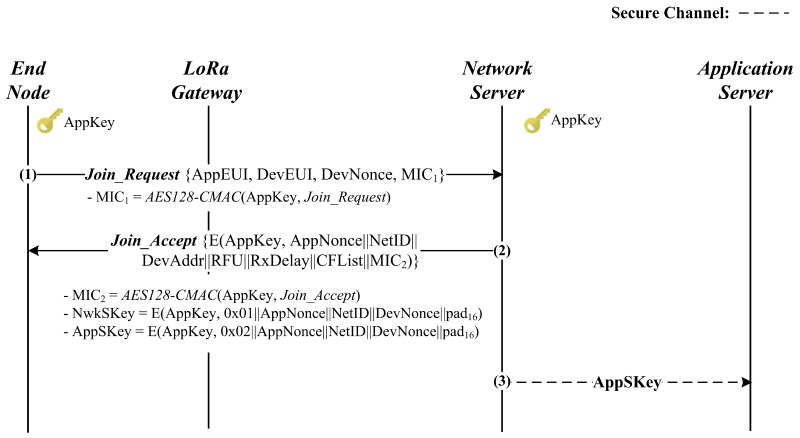
LoRaWAN Join procedure.

The join procedure is depicted in Figure 2. In order to enter the LoRaWAN network, the device starts the join procedure by sending the *Join_Request* message. Prior to this, it first randomly generates *DevNonce* and then computes $MIC_{1}$ with the long-term secret key *AppKey*. Upon receiving the message, the network server verifies the included $MIC_{1}$. If positive, it gains trust for the device and then randomly generates *AppNonce* to proceed to the next steps. Afterwards, the network server prepares for the *Join_Accept* message by computing $MIC_{2}$ and encrypting the message $AppNoncei||NetID||DevAddr||RFU||RxDelay||CFList||MIC_{2}$ with *AppKey*. At the same time, it makes the two session keys $AppSKey=E(AppKey,0X01||AppNonce||NetID||DevNonce||pad_{16})$ and $NwkSKey=E(AppKey,0X02||AppNonce||NetID||DevNonce||pad_{16})$. The network server concludes the join procedure by sending the *Join_Accept* message to the device and forwarding *AppSKey* to the application server. On receipt of the message, the device decrypts it and verifies the included $MIC_{2}$. If the verification is successful, the network server is authenticated to the device, which then generates the two session keys, *AppSKey* and *NwkSKey*. As a result, the device and the network server mutually authenticate each other while exchanging the two session keys. In addition, *AppSKey* is shared between the device and the application server.

### 2.3. Problems with the Standard LoRaWAN Protocol

The standard LoRaWAN protocol faces the following problems irrespective of its preliminary securities:

- There is no prevention against the replay attack in the *Join_Request* and *Join_Accept* messages because the device and the network server cannot accept the freshness of *AppNonce* and *DevNonce*, respectively.
- The end-to-end security between the device and the application server is broken because *AppSKey* is known to the network server.
- The session keys are derived from the long-term secret key *AppKey*. Therefore, if *AppKey* is compromised, the past session keys can be recovered while the encrypted traffic can be decrypted, i.e., the perfect forward secrecy does not hold.

## 3. Related Work

As mentioned above, because the network server generates two session keys, network operators are able to decrypt and intercept all the data passing through the network server. Girard [52] pointed out this problem and suggested to deploy the trusted third party to enhance the LoRaWAN network. According to the LoRaWAN specification v1.02 [51], it is clearly defined that compromising the keys of the one device does not impact the other ones’ secure communication. However, in ABP mode, the keys are derived from the device address, which leads to a vulnerability with reverse engineering [40]. Further, with the standard protocol, there exists a loophole of the end-to-end security and the vulnerability to the replay attack.

Avoine and Ferreira [53] introduced several attacks that affect the network availability, data integrity and data confidentiality in the earlier versions of LoRaWAN. The authors emphasized the replay or decrypt attack and desynchronization attack. These attacks are discussed by considering an end-device or the network server as the target entity. In the replay attack scenario, the authors discuss two techniques for attack: replay of a *Join_Accept* message and harvest of Join messages. Similarly, in the desynchronization attack, the target can be an end device or the network server, which is responsible for disconnecting the end-device from the network. By considering these attacks, the authors recommended that *AppNonce* value should follow freshness, provide the detection mechanism against the replay attacks, verify that the received *Join_Accept* message corresponds to the sent *Join_Request* message and also check that session keys are shared or not.

Kim and Song [42] tried to provide end-to-end security between the device and the application server by allowing the two parties to directly negotiate *AppSKey* without involving the network server. However, it needs to change the standard, which makes its application difficult in the existing LoRaWAN network. Moreover, this approach cannot provide the perfect forward secrecy.

Na et al. [41] introduced an effective countermeasure against the replay attack in the join procedure. In more detail, this approach uses eXclusive-OR *DevNonce* with *AppKey* or the previous session key to make the *Join_Request* message fresh. Even though this approach effectively addresses the replay attack, it does not support the end-to-end security, nor the perfect forward secrecy.

In Garcia et al. (radius-based) [44], an explicit entity termed as the join server is used, which is responsible for authentication of devices. The join server plays the role of an external Authentication, Authorization, and Accounting (AAA) server. In this approach, the AAA server instead of the network server mutually authenticates the device while exchanging the session keys. With the integration of the LoRaWAN joining procedure with the radius mechanism, the AAA server can make the network server free from the key management overheads. However, even in such a scenario, the network server knows all the keys, thus being able to decrypt all the packets between the device and the application server (i.e., the end-to-end security is broken). Moreover, a long latency is also caused because of the involvement of the AAA server. Further, this scheme does not focus on the replay attack problem in the join request.

In Garcia et al. (diameter-based) [43], the network server serves as the diameter client to communicate with the AAA server. On receiving the join request from the device, the network server counts on the AAA server to authenticate the device and generate the session keys. Similar to the radius-based approach, this approach can make the network server free from the key management overheads. However, because the network server knows all the keys, it can decrypt and understand all the packets between the device and the application server (i.e., the end-to-end security is broken). In addition, a long latency happens without a focus on the replay attack problem in the join request. A state-of-the-art comparison of different LoRaWAN schemes is presented in Table 2.

## 4. Enhanced LoRaWAN Security Protocol

This section presents the enhanced LoRaWAN security protocol, which addresses the standard one’s security flaws. It not only suggests the *DevNonce* generation method to prevent the replay attack, but also enables a device and its application server to achieve the true end-to-end security through the Elliptic Curve Diffie Hellman (ECDH)-based key exchange [54], which is authenticated by the Elliptic Curve Digital Signature Algorithm (ECDSA) [55]. Especially, the presented protocol provides two options, the Default Option (DO) and the Security-Enhanced Option (SEO), to prevent a malicious network server from breaking the end-to-end security between a device and its application server. The first option DO aims to defend against a malicious network server attempting to eavesdrop on the communication between a device and its application server. In the second option SEO, a malicious network server is blocked from manipulating packets between a device and its application server, as well as impersonating these two parties.

The proposed protocol has the following assumptions: (i) AppKey is a long-term secret shared between a device and its network server. (ii) A secure channel is pre-established between the network and application servers. (iii) Every device has and trusts its application server’s ECDSA public key $PU_{APP}$, which is used to verify the application server’s digital signature. (iv) In the case of SEO, every device should have its own ECDSA public key pair, and its public key $PU_{DEV}$ should be trusted by the application server. How $PU_{APP}$ and $PU_{DEV}$ are safely delivered, revoked and updated is beyond this paper’s scope. Here, the LoRa gateway is skipped because it has no contribution to security.

### 4.1. Default Option

**Figure 3:**
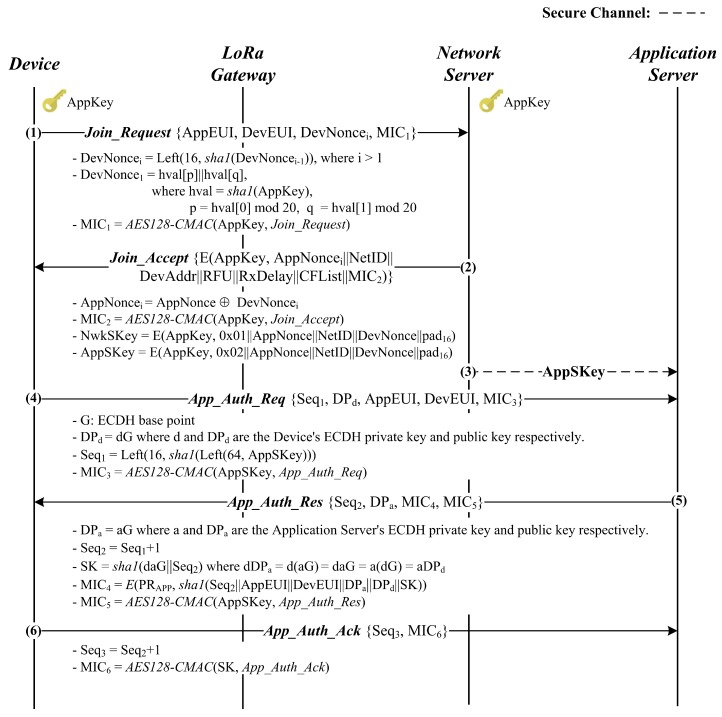
Proposed protocol’s default option.

Figure 3 outlines the first option DO, which is composed of six steps. In the first two steps, the device and the network server authenticate each other while exchanging two session keys. Then, the device and the application server perform mutual authentication, as well as establish the strong session key, SK, based on which, both the end-to-end security and the perfect forward secrecy hold.

Details on this option are described as follows.

**Figure 4:**
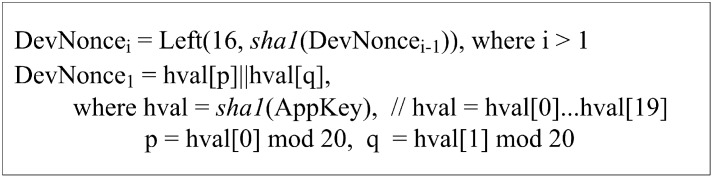
DevNonce generation.

(1) A device attempts to enter the LoRaWAN network by sending the *Join_Request* message. To protect this message, the device generates the *i*-th fresh nonce $DevNonce_{i}$ and the message integrity code $MIC_{1}$. Figure 4 shows how the $DevNonce_{i}$ is computed in the way of making the *Join_Request* message fresh, which enables this step to prevent replay attacks. In addition, $MIC_{1}$ is obtained by computing AES128−CMAC(*AppKey*, *Join_Request*).
(2)–(3) On receiving the *Join_Request* message, the network server verifies if the received $DevNonce_{i}$ and $MIC_{1}$ are correct. In the positive case, the device is successfully authenticated to the network server, which then prepares for the network server’s *i*-th nonce $AppNonce_{i}$ by eXclusive-ORing a randomly generated nonce AppNonce with the received $DevNonce_{i}$, generates two session keys AppSKey and NwkSKey and computes $MIC_{2}$. Note that $AppNonce_{i}$ can guarantee the device the *Join_Accept* message’s freshness and relation to the received *Join_Request* message. Finally, the network server encrypts with AppKey all the message contents including $AppNonce_{i}$, NetID, DevAddr, RFU, RxDelay, CFList and $MIC_{2}$ into the *Join_Accept* message, which is then sent to the device. At the same time, the network server forwards the application server the newly-generated session key AppSKey so that the *App_Auth_Req* message can be safely exchanged with that key. Upon a receipt of the *Join_Accept* message, the device decrypts it with AppKey and verifies the correctness of the decrypted $AppNonce_{i}$ and $MIC_{2}$. If the verification is successful, the device can conclude that the message is fresh and the network server is authentic, followed by computing the two session keys, AppSKey and NwkSKey. At this point, it is worth noting that this protocol provides the first two messages’ freshness, the mutual authentication between the device and the network server and the session key exchange.
(4) Once having successfully verified the *Join_Accept* message, the device proceeds with the remaining Steps (4)–(6) by preparing for the *App_Auth_Req* message. For this message, it first computes $Seq_{1}=\left(16,sha1\left(64,AppSKey\right)\right)$, which is made fresh by being derived from AppSKey. In addition, its ECDH private key d is randomly generated, and the corresponding public key $DP_{d}=dG$ is computed where G is the base point. Finally, the session key AppSKey is applied to compute $MIC_{3}=AES128-CMAC$(*AppSKey*, *App_Auth_Req*), which secures both the *App_Auth_Req* message and the ECDH key exchange.
(5) Upon receiving the *App_Auth_Req* message, the application server validates $Seq_{1}$ and $MIC_{3}$. If valid, it randomly creates its own ECDH private key a and calculates the corresponding public key $DP_{a}=aG$, followed by obtaining $SK=sha1(aDP_{d}||Seq_{2})$. At this point, the application server can defend against resource exhaustion attacks by flooding the *App_Auth_Req* messages because it first checks $MIC_{3}$ prior to the expensive ECDH operations. Afterwards, it makes $MIC_{4}=E(PR_{App},sha1(Seq_{2}||AppEUI||DevEUI||DP_{d}||DP_{a}\left||SK\right)$ and $MIC_{5}=AES128-CMAC$(*AppSKey*, *App_Auth_Res*), which, together with other values, constitute the *App_Auth_Res* message. Note that $MIC_{4}$, which is a digital signature generated with the application server’s private key $PR_{App}$, plays a role in defending against the man-in-the-middle attacks launched by a malicious network server. The application server finishes Step (5) by responding to the device with the *App_Auth_Res* message.
(6) After receiving the *App_Auth_Res* message, the device first verifies the received $Seq_{2}$ and $MIC_{5}$. If they are valid, the device trusts that the received message is fresh and protected by AppSKey. Such a trust allows the device to establish the session key SK without being vulnerable to the replay and resource exhaustion attacks. Then, the device validates $MIC_{4}$ with the application’s public key $PU_{APP}$. If $MIC_{4}$ is correct, the application server is authenticated to the device, which then concludes the proposed protocol by sending the *App_Auth_Ack* message. At this point, the device can prevent the man-in-the-middle attacks by a malicious network server because $MIC_{4}$ can only be generated by the application server. Once the message arrives, the application server attempts to verify if it is fresh and valid with the received $Seq_{3}$ and $MIC_{6}$. If this verification is successful, the device is authenticated to the application server, as well as shown to have SK.

It is worth noting that the first option DO counts on the digital signature $MIC_{4}$ to block a malicious network server from carrying out the man-in-the-middle attack on the ECDH-based key exchange. That makes it impossible for the malicious server to interpret and manipulate the packets transmitted between the device and the application server. However, this option is still vulnerable to the impersonation attack that the malicious network server can launch by forging the *App_Auth_Req* message with *AppSKey*.

### 4.2. Security-Enhanced Option

**Figure 5:**
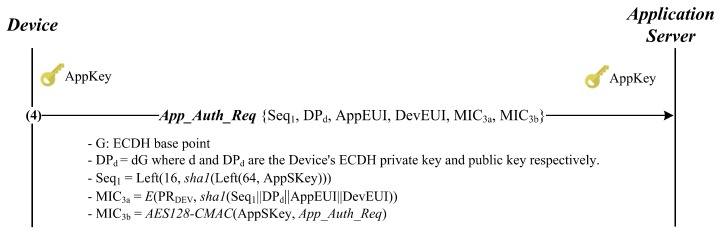
Proposed protocol’s security-enhanced option.

The second option SEO aims at defeating the impersonation attack mentioned above while keeping the DO’s security properties. Therefore, as shown in Figure 5, this option’s messages are exactly the same as those of DO except for the *App_Auth_Req* message. In order to prevent the impersonation attack by the malicious network server, the *App_Auth_Req* message includes the digital signature $MIC_{3a}$ generated with the device’s ECDSA private key, $PR_{DEV}$. This signature disables the network server to masquerade as the device by using *AppSKey*. Here, we assume that with the given *DevEUI*, the application server can securely retrieve the device’s ECDSA public key, $PU_{DEV}$, from its centralized repository rather than directly from the device.

## 5. Security Analysis

In this section, the proposed protocol is formally analyzed through BAN logic [10,46,56,57], and then, the possibility of attack is thoroughly examined by the AVISPA tool.

### 5.1. BAN Analysis

**Figure 6:**
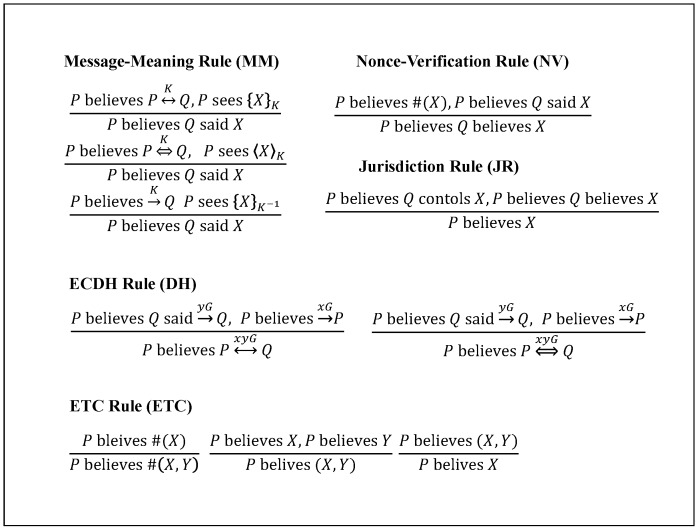
BAN logic rules.

According to the typical BAN logic analysis, the proposed protocol is first idealized; its assumptions and goals are defined, and then, the belief derivation is repeatedly conducted until obtaining the intended results. The BAN logic’s notations and rules are shown in Table 3 and Figure 6.

#### 5.1.1. Default Option

At first, the default option DO is translated into an idealization as follows (where *D*, NS and APP denote device, network server and application server, respectively): 
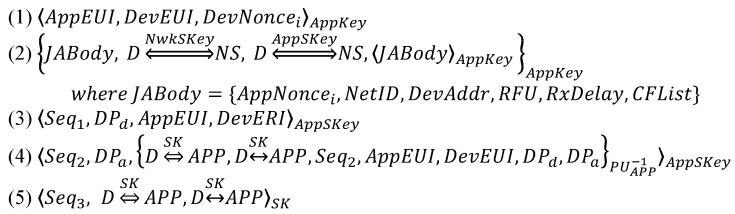


As the next step, it is necessary to make the DO’s assumptions and goals. The assumptions are as follows:
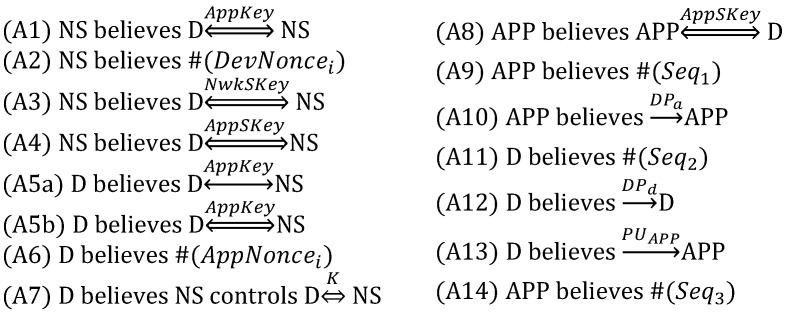


Strictly speaking, (A8) is not reasonable because *AppSKey* is known to the network server in addition to the device and the application server. However, DO does not consider the malicious network server trying to impersonate the device by forging the *App_Auth_Req* message. Therefore, (A8) is maintained to reason about DO under such an attacker model.

The goals are as follows:
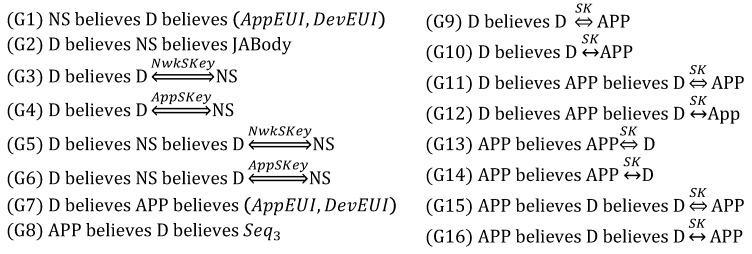


In the above goals, (G1) and (G2) indicate the mutual authentication between the device and the network server, while (G7) and (G8) indicate the mutual authentication between the device and the application server. On the other hand, (G3)–(G6) mean that the device obtains the belief that it shares the two session keys, *NwkSKey* and *AppSKey*, with the network server. In addition, the remaining goals express that the session key *SK* is securely exchanged between the device and the application server. With the above-idealized version, assumptions and goals, the formal analysis proceeds.

From (1), we derive:
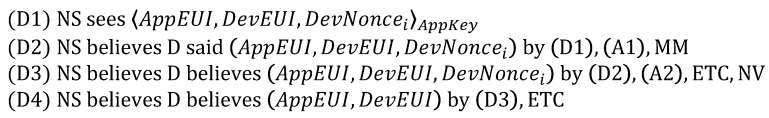


From (2), we derive:
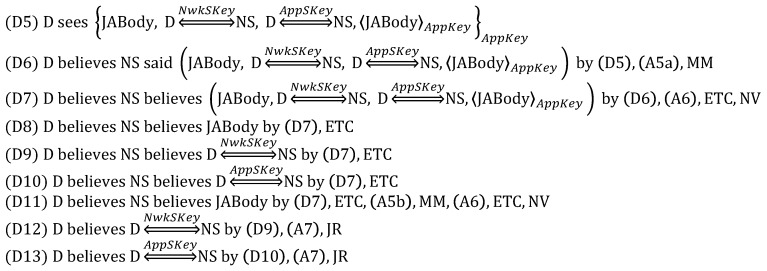


From (3), we derive:
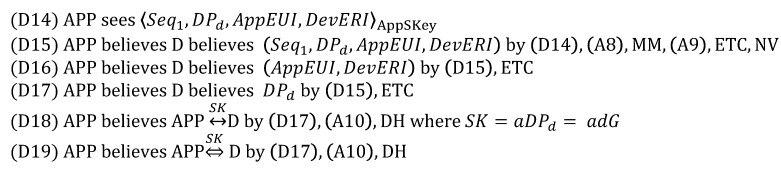


From (4), we derive:
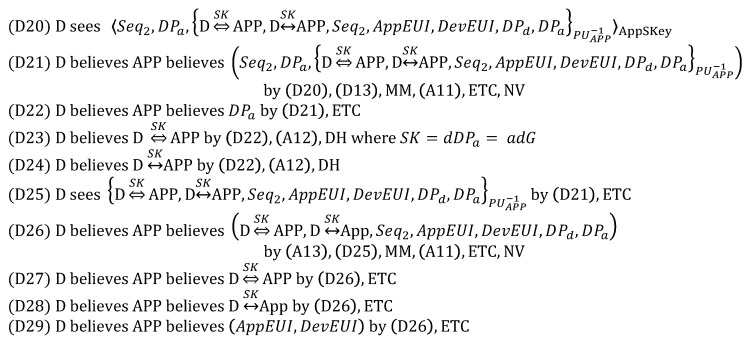


From (5), we derive:
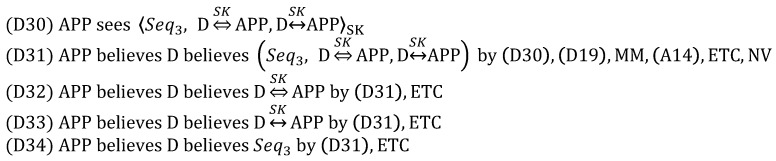


Based on the above derivations, we can show that the goals are satisfied as follows, while thus concluding that DO is correct.

- (G1) and (G2) are satisfied by (D4) and (D8), respectively.
- (G3)–(G6) are satisfied by (D12), (D13), (D9) and (D10), respectively.
- (G7) and (G8) are satisfied by (D29) and (D34), respectively.
- (G9)–(G16) are satisfied by (D23), (D24), (D27), (D28), (D18), (D19), (D32) and (D33), respectively.

#### 5.1.2. Security-Enhanced Option

In order to analyze SEO, we focus on only the *App_Auth_Req* message because DO and SEO are the same except for it. Therefore, the *App_Auth_Req* message is idealized as follows:
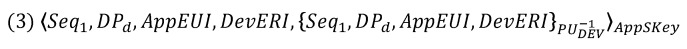


The assumption (A15) and the goal (G17) are added as follows:
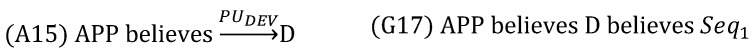


Here, like (G8), (G17) indicates that the device is authenticated to the application server. In other words, (G8) is complemented by (G17), which is obtained through ECDSA.

From (3), we derive:
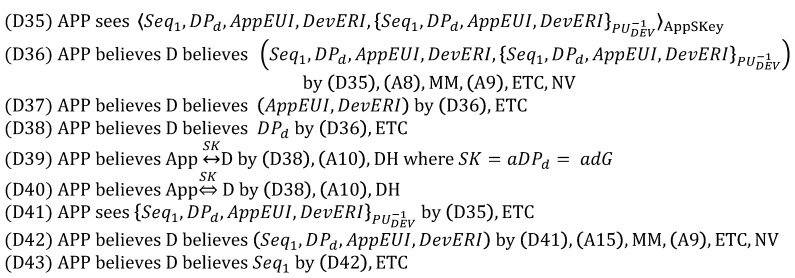


Obviously, the goal (G17) is achieved by obtaining (D43), while (G13) and (G14) are satisfied through D(39) and (D40). Unlike DO, SEO aims to defeat the malicious network server trying to masquerade as the device with *AppSKey*. For this goal, the device is authenticated to the application server by using the digital signature $MIC_{3a}$ computed with the ECDSA private key $PR_{DEV}$. Moreover, *AppSKey* plays a role in preventing the resource exhaustion attack by allowing the application server to check $MIC_{3b}$ prior to the expensive operations, i.e., ECDSA digital signature verification and ECDH key exchange. Thus, it is still reasonable to maintain (A8). As a result, we can conclude that SEO is correct.

#### 5.1.3. Security Properties

Mutual authentication: The proposed protocol provides the two mutual authentications, i.e., the one between the device and the network server and the other between the device and the application server. Here, by presenting the two lemmas, Lemma 1 and Lemma 2, we prove that the two mutual authentications hold in the proposed protocol.

**Lemma** **1.** *The device and network server mutually authenticate each other.*

**Proof.**  It is demonstrated from (D4) and (D11) that the device and the application authenticate each other. Therefore, the proposed protocol satisfies the mutual authentication between the device and the application server. ☐

**Lemma** **2.** *The device and application server mutually authenticate each other.*

**Proof.**  This mutual authentication needs to be proved considering the two options DO and SEO. In the DO case, the proof relies on (D29) and (D34) to show that the device and the application authenticate each other. Note that DO allows (A8) and (D17) because of not taking into consideration the malicious network server impersonating a device. Consequently, the mutual authentication is achieved in DO. On the other hand, in the SEO case, it can be shown from (D29) and (D43) that the mutual authentication between the two parties is satisfied. Clearly, these two obtained beliefs indicate that it is impossible for the malicious network server to launch the man-in-the-middle and impersonate attacks and break the end-to-end security. As a result, it is concluded that the proposed protocol achieves the mutual authentication between the device and the application server. ☐

Secure key exchange: In the proposed protocol, three session keys including *NwkSKey*, AppSKey and SK are exchanged where *NwkSKey* is shared between the device and the network server, as well as both *AppSKey* and SK are shared between the device and the application server. Here, we provide Lemma 3 and Lemma 4 to prove that these keys are securely exchanged.

**Lemma** **3.** *NwkSKey and AppSKey are securely exchanged between the device and network server.*

**Proof.**  Based on (D9), (D10), (D12) and (D13), we can verify that the session key exchange is authenticated to the device. On the other hand, it can be shown from (D4), (A3) and (A4) that the network server validates the session key exchange. As a result, we can reason that NwkSKey and AppSKey are securely exchanged between the two parties. Note that once the session key exchange is successfully performed, *AppSKey* is forwarded to the application server. Thus, *AppSKey* is finally shared between the device and the application server. This session key is properly used according to the selected option, i.e., DO or SEO, so that the security threats caused by the malicious network server can be avoided. ☐

**Lemma** **4.** *SK is securely exchanged between the device and application server.*

**Proof.**  In the DO case, it can be seen from (D18), (D19), (D23), (D24), (D27), (D28), (D32) and (D33) that *SK* is securely exchanged between the device and the application server. In the SEO case, the secure key exchange between the two parties can be verified based on (D23), (D24), (D27), (D28), (D32), (D33), (D39) and (D40). Consequently, we can conclude that *SK* is securely established between the device and the application server. ☐

End-to-end security: In the proposed protocol, it is very important to provide the end-to-end security between the device and the application server. *AppSKey*, which aims to protect the application traffic, cannot keep this security property because it is known to the network server in addition to the two involved parties. To solve this problem, the proposed protocol allows the device and the application server to securely share the session key *SK* by relying on the ECDH-based key exchange and the ECDSA digital signature. Lemma 5 is proposed to prove that the proposed protocol achieves the end-to-end security between the device and the application server.

**Lemma** **5.** *The end-to-end security is provided between the device and application server.*

**Proof.**  In the DO case, it is shown from (D18), (D19), (D23) and (D24) that *SK* is established between the device and the application server via the ECDH-based key exchange. Especially, (D17) and (D29) validate that this key exchange can be trusted. Similarly, in the SEO case, (D23), (D24), (D39) and (D40) verify that the device and the application share *SK* by the ECDH-based key exchange. Such a key exchange is secured according to (D29) and (D43). As mentioned above, *SK* is exchanged in a way that it is only known by the two parties involved through the ECDH-based key exchange. In other words, the network server cannot decrypt or modify the application traffic once it is encrypted or protected with the session keys derived from *SK*. Therefore, it can be concluded that the end-to-end security is offered between the device and the application server. ☐

Perfect forward secrecy: Perfect forward secrecy is a security property where the compromise of long-term keys does not cause the past session keys to be exposed [58]. For this property, we take into consideration *SK* because it is used to encrypt the application traffic. Below, we provide Lemma 6 to testify that the proposed protocol achieves perfect forward secrecy on the application traffic.

**Lemma** **6.** *Perfect forward secrecy is provided for SK.*

**Proof.**  According to the obtained beliefs (D18), (D19), (D23), (D24), (D39) and (D40), it is verified that *SK* is securely exchanged between the device and the application server via the ECDH-based key exchange. In addition, we can see from (D17), (D29) and (D43) that the *SK* exchange is protected through the ECDSA digital signature, thus defending against the man-in-the-middle attack. It is worth noting that the generated ECDH private keys are forgotten after their session. That makes it impossible for the past session keys to be recovered even when long-term keys are compromised. Therefore, it is proven that perfect forward secrecy is provided for *SK*. ☐

Defense against resource exhaustion attack: Here, we provide Lemma 7 to show that the proposed protocol prevents the resource exhaustion attack, which sends many messages requesting the expensive public key operations to cause the application server and the device to uselessly waste their resources.

**Lemma** **7.** *The proposed protocol defends against resource exhaustion attack.*

**Proof.**  In the proposed protocol, the application server checks $MIC_{3}$ or $MIC_{3}b$ prior to the expensive public key operations. Similarly, the device verifies the digital signature after validating $MIC_{5}$. Therefore, from the derived beliefs (D17), (D22) and (D38), it is demonstrated that the proposed protocol defends against the resource exhaustion attack. ☐

Defense against replay attack: In the proposed protocol, $DevNonce_{i}$ is made fresh according to the *DevNonce* generation method, while $AppNonce_{i}$ is made fresh by eXclusive-ORing the randomly-generated nonce *AppNonce* with $DevNonce_{i}$. Thus, by including these fresh values, the *Join_Request* and *Join_Accept* messages are not vulnerable to the replay attack. On the other hand, the *App_Auth_Req*, *App_Auth_Res* and *App_Auth_Ack* messages depend on the sequence numbers $Sep_{1}$, $Sep_{2}$ and $Sep_{3}$ to prevent the replay attack. Especially, $Sep_{1}$ is guaranteed to be fresh because it is derived from the new session key *AppSKey*, and the subsequent numbers are generated by increasing their previous number by one. As a result, the proposed protocol is not vulnerable to the replay attack.

### 5.2. AVISPA Analysis

**Figure 7:**
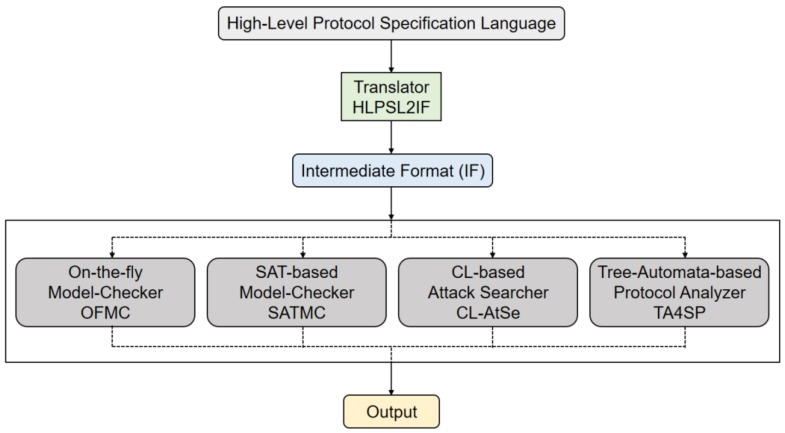
Architecture of Automated Validation of Internet Security Protocols and Applications (AVISPA).

AVISPA [47] is an automatic tool for modeling and analyzing security protocols. In AVISPA, a security protocol is modeled based on the High-Level Protocol Specification Language (HLPSL) [59] and converted via HLPSL2IF to Intermediate Format (IF). The converted IF version is then formally analyzed through four sub-modules as shown in Figure 7, and the result is derived.

The four sub-modules are as follows:

- OFMC: On-the-Fly Model-Checker [60]
- SATMC: SAT-based Model-Checker [61]
- CL-AtSe: CL-based Attack Searcher [62]
- TA4SP: Tree-Automata-based Protocol Analyzer [63]

#### 5.2.1. Default Option

The proposed protocol’s DO is modeled in the HLPSL version, which is composed of three roles: the basic, composed and environment roles.

**Figure 8:**
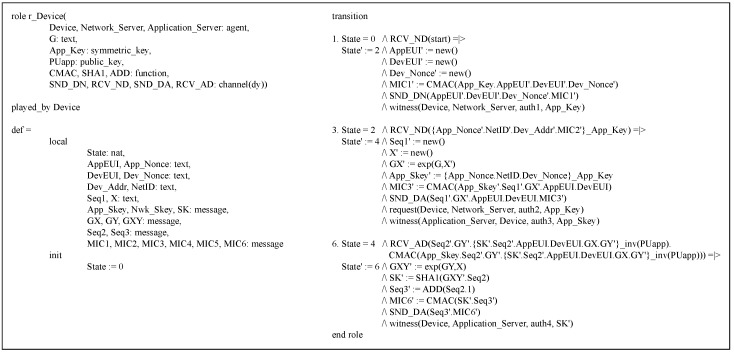
r_Device role.

Basic role: The DO’s HLPSL version includes the three basic roles, r_Device, r_Network_Server and r_Application_Server, which correspond to a device, network server and application server, respectively. Figure 8 shows the source code of the device’s basic role r_Device.

This role possesses the shared key *App_Key* and its application server’s public key *PUapp*. Furthermore, it communicates with r_Network_Server using the SND_DN and RCV_ND channels while communicating with r_Application_Server using the SND_DA and RCV_AD channels. Note that all the basic roles including r_Device apply the Dolev–Yao (dy) model, one of the attacker models, to the channels. The basic operations of r_Device are defined in the transition section.

**Figure 9:**
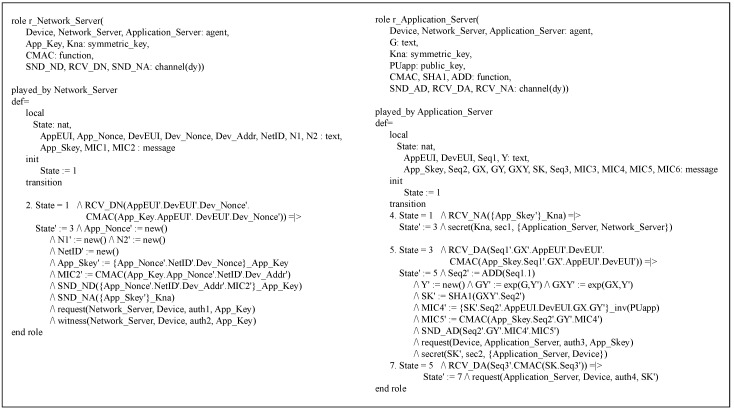
r_Network_Server role and r_Application_Server role.

In Figure 9, r_Network_Server is defined as a basic role to model the network server. This role shares *App_Key* and *Kna* with r_Device and r_Application_Server, respectively. Especially, *Kna* is used to protect the channel SND_NA between r_Network_Server and r_Application_Server. Moreover, the SND_ND and RCV_DN channels are defined for communication between r_Device and r_Network_Sever. How r_Network_Server works is described in detail in the transition section. The last basic role r_Application_Sever is expressed for the application server as shown in Figure 9. With the symmetric key *Kna*, this role is able to securely communicate with r_Network_Server over the channel RCV_NA. In addition, the private key corresponding to *PUapp*, which is expressed as inv(*PUapp*), is utilized to calculate a digital signature. The detailed operations of r_Application_Sever are specified in the transition section.

**Figure 10:**
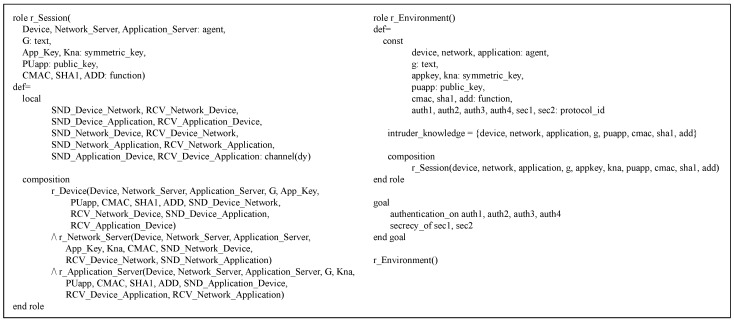
r_Session role and r_Environment role.

Composed role and environment role: Figure 10 illustrates the composed role of the HLPSL model, r_Session, which represents a session of the proposed protocol.

After declaring all channels, r_Session arranges and calls three basic roles with necessary parameters to express an entire session. The r_Environment role is specified for the proposed protocol in Figure 10, which defines important constants for agents, keys, functions, etc, sets the attacker’s knowledge and decides how the proposed protocol executes. Moreover, this role makes the security goals that the proposed protocol should satisfy. In more detail, the four goals, auth1, auth2, auth3 and auth4, are defined to check if the proposed protocol’s authentication holds, followed by the two goals sec1 and sec2, defined for confirming the proposed protocol’s secrecy.

**Figure 11:**
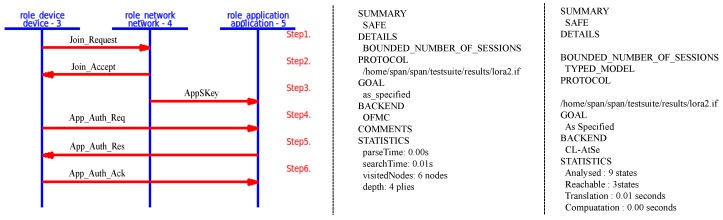
DO simulation and analysis result.

Result: Figure 11 shows how the proposed protocol runs in the AVISPA environments. Furthermore, in this figure, we can see the formal verification results on the proposed protocol obtained through the sub-modules of OFCM and CL-AtSe, respectively. According to the results, we can confirm that there is no attack.

#### 5.2.2. Security Enhanced Option

**Figure 12:**
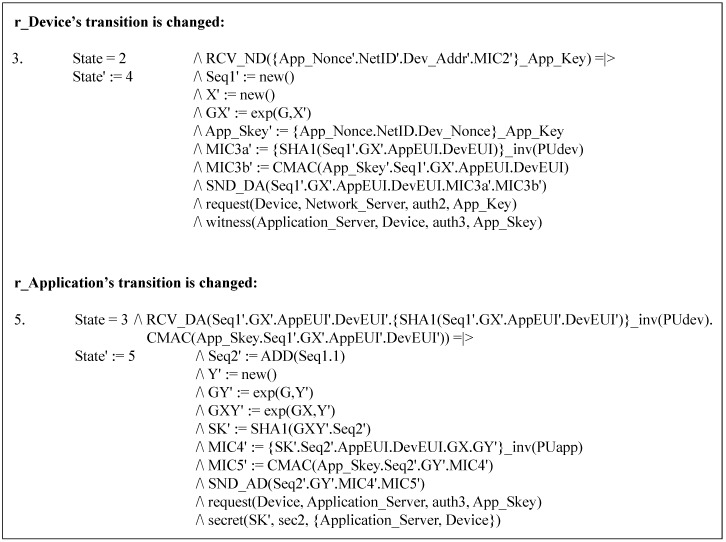
Major changes in the Security-Enhanced Option (SEO).

In SEO, the DO’s roles are modified to model the device’s digital signature $MIC_{3a}$ and its related operations. As depicted in Figure 12, the major changes are made in the transitions of r_Device and r_Application. The SEO’s simulation and analysis results are exactly the same as those of DO, indicating that no attack is found.

## 6. Performance Evaluation

Initial evaluations are conducted to calculate the message size and the overheads involved in the proposed protocol during communication between the end-to-end devices through coding over real hardware. Next, the two options of the proposed protocol, DO and SEO, are analyzed for their performance by using numerical simulations in comparison with the DTLS-PSK and DTLS-ECC protocols through a case study on a smart factory-enabled parking system.

**Figure 13:**
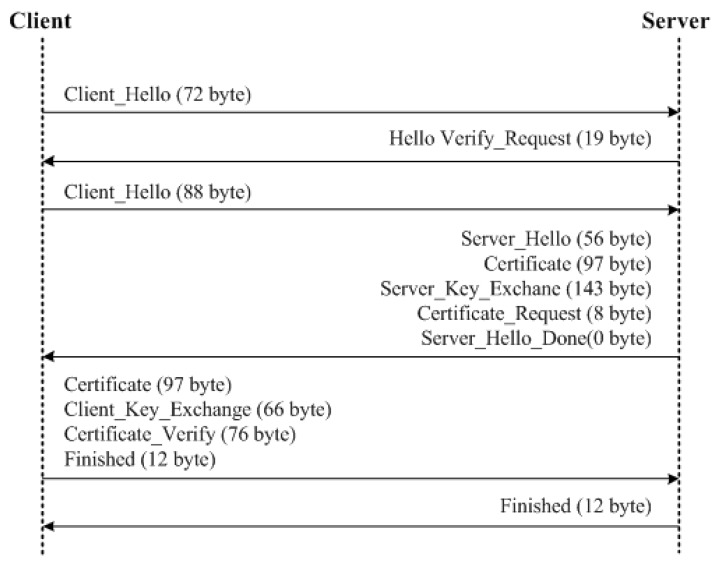
DTLS-ECC.

**Figure 14:**
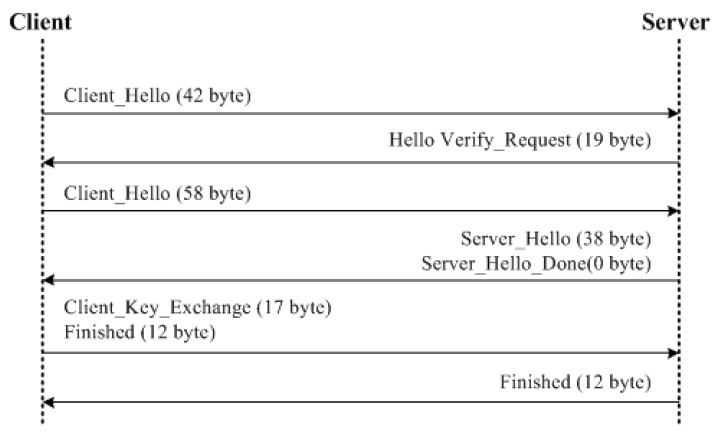
DTLS-PSK.

Figure 13 and Figure 14 illustrate the communication scenario between a client and a server for DTLS-PSK and DTLS-ECC, respectively. Both DTLS protocols are evaluated using the TinyDTLS 0.8.2 library with their respective cipher suite over the system with configurations presented in Table 4.

DTLS-PSK uses client and server initiations along with the exchange messages followed by the finish mechanisms for handshake during a contact between the client and the server. The client messages for DTLS-PSK cause an overhead of 1 ms for each operation, whereas there is no overhead due to server-initiation. Here, overheads are measured as the difference between the time after sending a handshake message and the time utilized before generating a handshake message. The server-overhead is only caused by finishing the communication. This lesser dependence on the server causes fewer overheads on the communication setup, but with compromised security. The details of packet size and overheads for DTLS-PSK are provided in Table 5. Using each packet size, the total message size used for handshaking between the client and the server is calculated, as shown in Table 6. From these system-based evaluations, it is obtained that DTLS-PSK causes a total overhead of 5 ms with a total message size of 198 bytes.

DTLS-ECC uses similar policies for a handshake as used by DTLS-PSK, but with the involvement of certification procedures for enhanced security of communication between the client and the server. The overhead scenarios are also similar to DTLS-PSK, i.e., 1 ms, but excessive overheads are caused due to the certificate exchange and verification procedures that raise the overhead to 31 ms. The details of total overhead for various procedures involved in the communication between the client and the server by using DTLS-ECC are presented in Table 7. Because of the certification scheme, DTLS-ECC uses a heavy packet size for a handshake between the involved entities. The maximum size packet is used for certificate-exchange procedures, which is 304 bytes, and the total message size reaches up to 746 bytes, as shown in Table 8.

Similarly to DTLS-PSK and DTLS-ECC, it is required to calculate the system-based message size and overhead caused by the proposed protocol for its implementation in practical scenarios. The environment setup for system-based evaluations of the proposed protocol is presented in Table 9. The proposed extended LoRaWAN protocol is evaluated for both its options. In DO, the overhead due to request and acknowledgment is 1 ms, and that of response is 9 ms. The message size varies between 37 and 53 bytes for request and response, respectively. The acknowledgment uses 4 bytes for its procedures. Thus, the total message size and the overhead for the proposed protocol in DO are 94 bytes and 11 ms, respectively, as shown in Table 10. In SEO of the proposed protocol, a malicious network server is blocked from manipulating packets between a device and its application server along with impersonating them. Thus, SEO consumes more time in generating final responses. Overall, the mechanism is not much different, but the extra features increase the message size of the SEO approach. The system-based evaluation for the proposed approach in SEO suggests that the total message size reaches up to 126 bytes, and the total overhead is 16 ms, as shown in Table 11. A comparison is drawn in Table 12 to understand the impact on the system-based evaluation for DTLS-PSK, DTLS-ECC and extended LoRaWAN protocol with DO and SEO.

### 6.1. Evaluation of the Proposed Protocol over a Low-Power Device

The system-based evaluations in the previous subsection help to determine the performance of the proposed protocol in a generic environment. However, the devices involved as end users in a LoRaWAN setup are of low-power ratings, such as mobiles, that may cause excessive overheads because of a difference in the type of CPU. To understand this variation, the two options, DO and SEO, of the proposed protocols are tested on a mobile platform with configurations given in Table 13. From the configurations, it can be identified that the CPU is limited in processing compared with system-based evaluations. The two options of the proposed protocol are coded separately for the Android version, and the results are recorded for overheads. The evaluations suggest that the proposed protocol with DO causes overhead in the range between 18 and 22 ms with a variation of 18.18%, whereas the proposed protocol with SEO causes overhead in the range between 26 and 33 ms with a variation of 21.21%, as shown in Table 14. The results for overhead are low even for a low-power device, which illustrates significant improvements in terms of security without affecting the performance.

### 6.2. Smart Factory-Enabled Parking System with the Proposed Protocol

**Figure 15:**
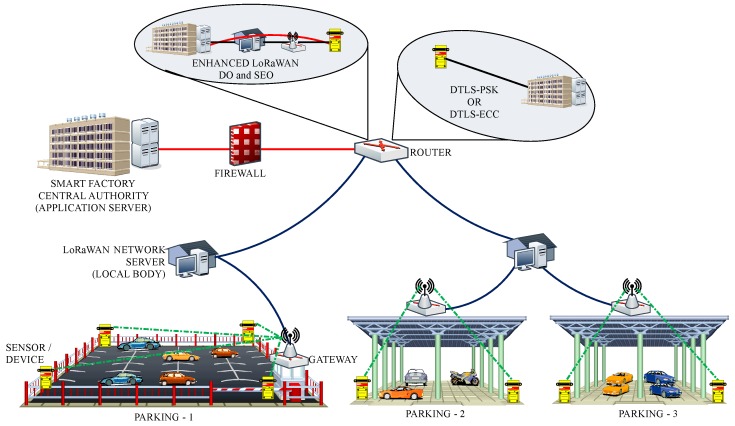
An illustration of the case study of protocols for a smart factory-enabled parking system.

Further, in order to understand the behavior of the proposed protocol, a smart parking scenario is considered, as shown in Figure 15. The scenario is comprised of multiple parking spaces each equipped with sensors, which transmit traffic to a local body via a gateway. The local body provides support for the LoRa network server, which is connected to the application server that is placed at the central authority. This case study can be matched with a special case, where a smart factory wants to track all the processes and the available parking places across the state. This information can be passed directly to the intended users via particular applications. On the contrary, the application servers can be replaced with direct communication with the users. However, in such a case, the authentication of the user is also to be considered, which may cause excessive overheads. Nevertheless, considering the scenario illustrated in this paper, the results are recorded for network latency and signaling overheads.

Irrespective of the communication, the traffic in the considered smart factory-enabled parking scenario passes through a series of wired, as well as wireless links. Thus, network latency is calculated by considering a single wired and a single wireless link in the network. Since the traffic remains the same, a similar message size, as computed previously, is used for evaluating the overall latency of the network. Thus, in this scenario, the network latency is calculated as [64]:

(1) $$N_{L}^{\left(O\right)}=T_{d}+\left(\frac{M}{F}-1\right)I+L2+\left(\frac{MK}{B}+W_{d}\right),$$

where $T_{d}$ is the one-frame transport delay, M is the message size, F is the frame size, I is the inter-frame time, L2 is the link layer delay, K is the number of intermediate hops, B is the network bandwidth and $W_{d}$ is the wired delay.

Next, the signaling overheads are calculated for the proposed approach by following host-based principles as the devices or the sensors are responsible for initiating the communication in the proposed setup. Thus, signaling overhead is given as [65]:

(2) $$S_{S}^{O}=\frac{KM}{\tau },$$

where τ is the session time between the involved entities. In case the network includes a master parking sensor, which manages all the sensors in the parking lot, the signaling overhead is calculated as:

(3) $$S_{M}^{O}=\frac{KM}{\tau }+\frac{(N-1)KM}{\tau },$$

where N is the number of sub-sensors in the parking lot. The details of values for each of the parameters used in the above formulations are given in Table 15.

**Figure 16:**
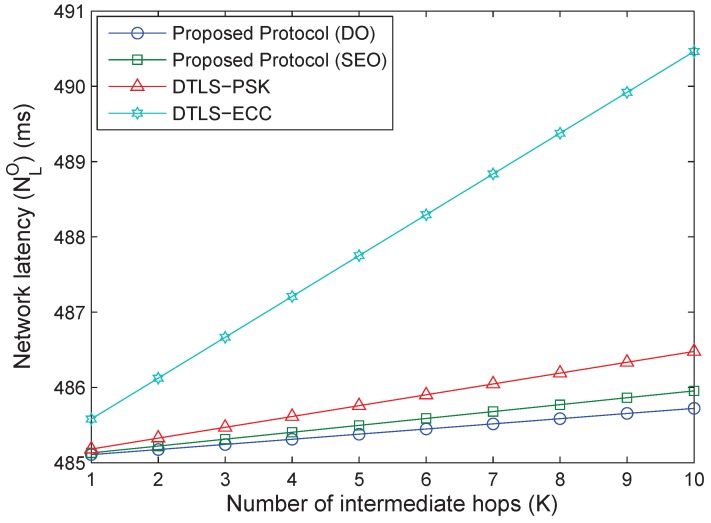
Network latency (ms) vs. the number of intermediate hops.

**Figure 17:**
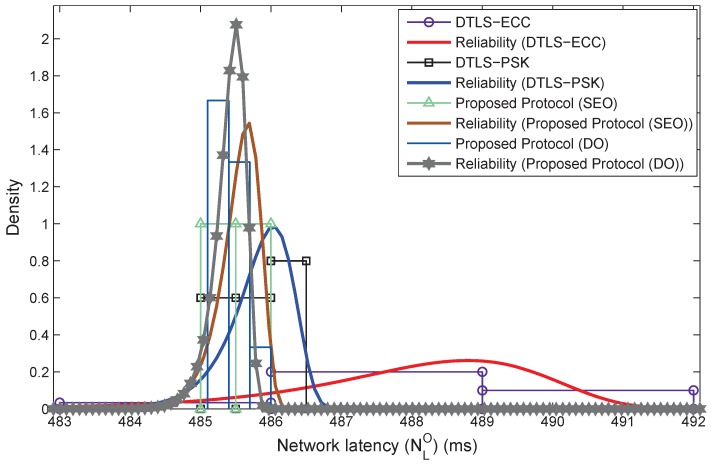
Density (network latency) vs. network latency with reliability fitting.

The proposed protocol operates with a maximum network latency of 485.4 and 485.5 ms for DO and SEO, respectively, whereas DTLS-PSK and DTLS-ECC operate with a maximum latency of 485.8 and 488.0 ms, respectively, with variation in the number of intermediate hops. The results in Figure 16 show an enhanced performance of DO and SEO for the given smart parking scenario. The results for network latency are evaluated over Weibull fitting for understanding the reliability of the proposed enhanced LoRaWAN protocol in comparison with the DTLS-PSK and DTLS-ECC through MATLAB™, as shown in Figure 17. The reliability curves suggest that the proposed protocol performs better and offers a higher reliability of communication for the smart factory-enabled parking system. These results are dominated by the message size, and the overhead study presented in earlier parts plays a crucial role in deciding these outputs.

**Figure 18:**
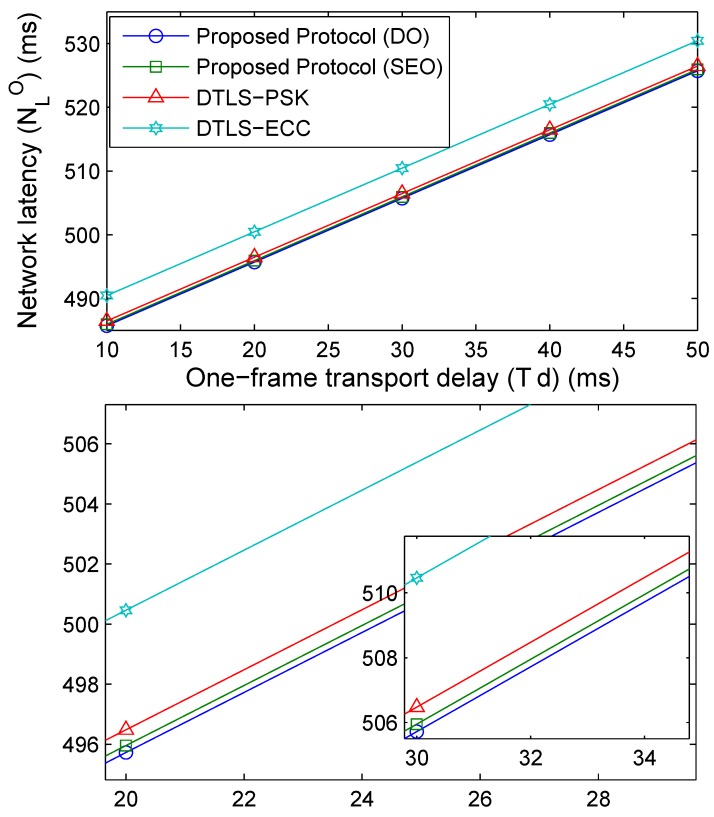
Network latency (ms) vs. transport delay.

Network latency is also affected by the frame-delays over the wired links. This can be seen in Figure 18, which shows an increasing trend for all the protocols with an increase in the one-frame transport delay. Further, DTLS-ECC suffers from the highest latency because of the certification procedures that result in the higher message size. The average network latency for the proposed protocol with DO, the proposed protocol with SEO, DTLS-PSK and DTLS-ECC is 505.7, 505.9, 506.4 and 510.4 ms, respectively.

**Figure 19:**
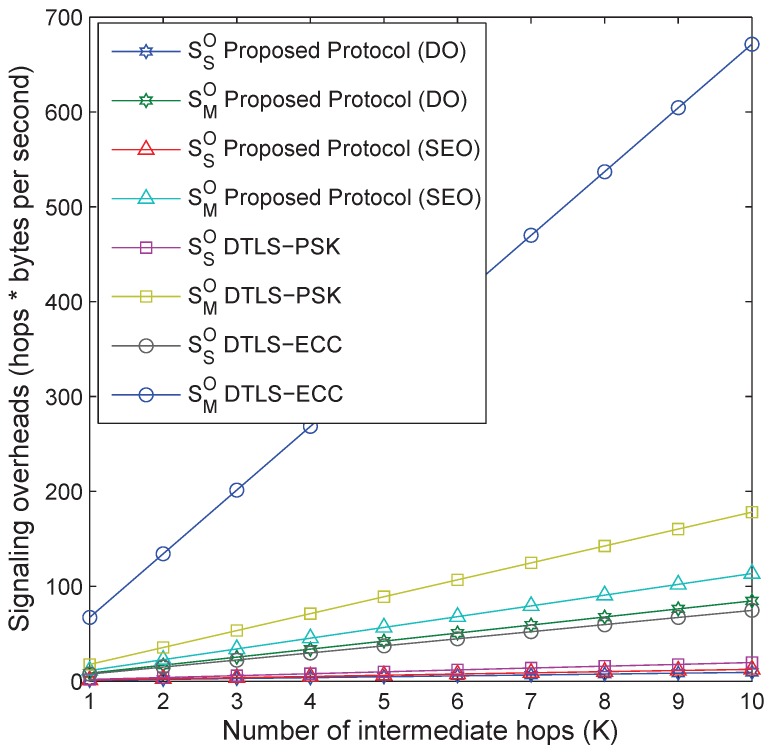
Signaling overheads vs. number of intermediate hops.

Finally, results are recorded for signaling overheads by following (2) and (3), as shown in Figure 19. These results are recorded for two sub-scenarios in the considered smart parking case study. Firstly, the results include signaling overheads ($S_{S}^{O}$) by using one-to-one communication between the application server and the single sensor, and secondly, the results include signaling overheads ($S_{M}^{O}$) by using one-to-one communication between the application server and the single master sensor, which facilitates multiple sensors of a particular parking lot. The results show that the proposed protocol in DO causes 52.5% and 87.3% fewer signaling overheads than DTLS-PSK and DTLS-ECC, respectively; and the proposed protocol in SEO causes 36.3% and 83.1% fewer signaling overheads than DTLS-PSK and DTLS-ECC, respectively.

### 6.3. Applicability to the New LoRaWAN Specification v1.1

**Figure 20:**
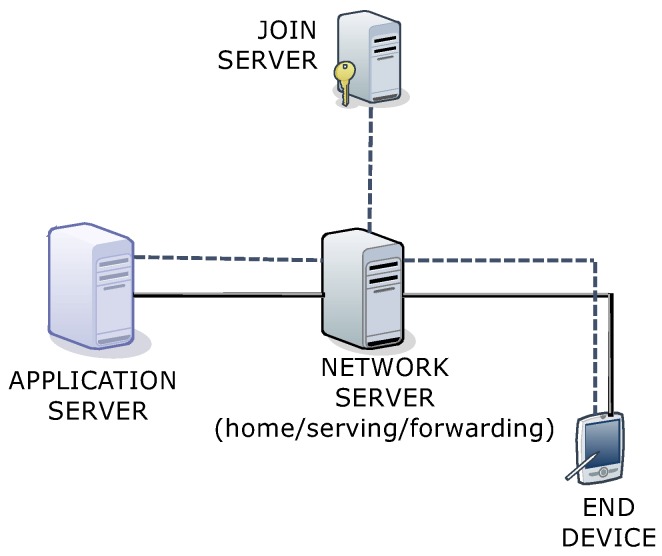
A general overview of the LoRaWAN specification v1.1 architecture.

Recently, a new LoRaWAN specification v1.1, as shown in Figure 20, has been released, which covers the majority of the shortcomings of the LoRaWAN specification v1.02. The new specification comprises similar components as that of the v1.02 specification except that network server’s functionalities are divided into home, serving and forwarding entities with security dependence on the join server. In the LoRaWAN specification v1.1, the data transferred between the application server and the end device are encrypted using the keys generated by the join server. Here, the involved network server aggregation is considered as trusted; however, a malicious network server may be able to alter the content of the data messages in transit, which may even help the compromised network server to infer some information about the data by observing the reaction of the application end-points to the altered data. Therefore, end-to-end security is needed between the application server and the end device, through which the data transmission can be protected in terms of confidentiality and integrity, and not compromised by the network server or any other entity.

These issues are further highlighted in the official release of the LoRa (https://www.lora-alliance.org/lorawan-for-developers), Alliance™, as well as by the organizations (http://iotdesign.embedded-computing.com/articles/lora-networks-in-buildings-reduce-infrastructure-costs/; https://www.businesswire.com/news/home/20180220005492/en/Cypress-ESCRYPT-Unveil-End-to-end-LoRaWAN-based-Security-Solution; https://micromaxtechnology.com/wp-content/uploads/Gemalto-IoT-LoRaWAN-Brochure.pdf; https://www.escrypt.com/en/solutions/secure-lorawan-communications) working on LoRaWAN applications. These detailed reports have clarified that the new specification assumes trust for network servers, but true end-to-end confidentiality and integrity protection are not yet covered by this new specification. Effective and secure strategic solutions are required for supporting the applications that have such requirements as their primary concern. All of these are supported by the proposed protocol that can secure the communications for both the LoRaWAN specifications v1.02 and v1.1.

It is to be noted that in the proposed protocol, DO focuses on preventing the passive attacks by the malicious network server, and SEO focuses on the active attacks by the malicious network server. Therefore, depending on the security requirements for the target service, a proper option can be selected for the LoRaWAN networks. The analyses presented in this paper by using BAN logic, the AVISPA tool, system simulations and the smart-parking case study demonstrate the performance of the proposed enhanced LoRaWAN protocol with the capability of securing the end-to-end communication between the device and the application server.

## 7. Conclusions and Future Remarks

Urban networks are largely affected by the need for smart applications, such as healthcare, city management, smart transportation and smart industry. Such networks are in demand, and with IoT, their applications can easily be extended across cities in the form of smart projects. In recent years, smart applications in the Internet of Things (IoT) were applied through a low-power communication setup, as the majority of devices in these networks are battery operated and smart applications running on them consume most of the battery life. One such facility is provided by the Low-Power Wide Area Network (LPWAN), but at a constrained bit rate. For facilitating long-range communication over LPWAN, several approaches and protocols are provided by different researchers and research organizations across the globe.

One of the popular protocols is the Long Range Wide Area Network (LoRaWAN), which is a media access layer protocol for long-range communication between the devices and the application servers via LPWAN gateways. However, LoRaWAN comes with issues related to security as a much-secured protocol will consume the majority of the resources (battery life) because of the excessive computational overheads and signaling cost. This may lead to several types of attacks on the network. The standard protocol fails to support the perfect forward secrecy, the end-to-end security and the defense against the replay attack. Thus, considering this as a problem, an enhanced LoRaWAN security protocol was proposed in this paper, which not only provides the functionalities of the basic protocol, but also prevents against different security threats.

The proposed protocol was developed with two options, the Default Option (DO) and the Security-Enhanced Option (SEO), to prevent a malicious network server from breaking the end-to-end security between a device and its application server. The initial security validations were conducted through the Burrows–Abadi–Needham (BAN) logic and the Automated Validation of Internet Security Protocols and Applications (AVISPA) tool. Next, system-based and low-power device-based performances were evaluated to understand the message size and the overhead of the proposed protocol. Further, for practical applications, the problem of a smart factory-enabled parking system was considered for secure and efficient parking management in smart cities. The results, in terms of network latency with reliability fitting and signaling overheads, show significant improvements and better performance for the proposed protocol in comparison with other security protocols, namely Datagram Transport Layer Security-Pre-Shared Key (DTLS-PSK) and Datagram Transport Layer Security-Elliptic Curve Cryptography (DTLS-ECC).

It is to be noted that the new LoRaWAN specification v1.1 is unable to support the end-to-end security between the user and the application server. This shortcoming can be overcome through our protocol, but details on the hardware-based implementation of the proposed protocol for LoRaWAN specification v1.1 will be presented in our future reports.

## Figures and Tables

**Table 1:** Notations table.

Symbol	Description
Join_request	Join request to attach the end device to the LoRa network
AppEUI	Application identifier
DevEUI	Device identifier
DevNonce	Nonce value randomly generated by the device
$DevNonce_{i}$	The *i*-th nonce value computed by the device
AES128−CMAC(K,M)	AES 128 cipher-based MAC function with the secret key *K* and the message *M*
MIC	Message integrity code
$MIC_{i}$	The *i*-th message integrity code except for the digital signatures $MIC_{3a}$ and $MIC_{4}$
NwkSKey	Network session key
AppSKey	Application session key
AppKey	The long-term key shard between a device and a network server
AppNonce	Nonce value randomly generated by the network server
$AppNonce_{i}$	The *i*-th nonce value computed by the network server
NetID	Network identifier
DevAddr	End device address
RxDelay	Delay between RX and TX
CFList	Optional list for channel frequencies
$sha_{1}\left(M\right)$	SHA 1 hash function, which takes an input *M* and produces a 160-bit
*App_Auth_Req*	Application authentication request message
*App_Auth_Res*	Application authentication response message
$Seq_{i}$	The *i*-th sequence number
SK	The session key between a device and its application server
||	Concatenation operation
*App_Auth_Ack*	Application authentication acknowledgment message
$PR_{APP}$	Private key of the application server
$PU_{APP}$	Public key of the application server
*a* and $DP_{a}$	Device’s elliptic curve Diffie–Hellman private and public keys
*b* and $DP_{b}$	Application server’s elliptic curve Diffie–Hellman private and public keys
*G*	Elliptic curve Diffie–Hellman base point
$pad_{16}$	Function adding zero octets to make the length of the data a multiple of 16
hval[.]	hval refers to hash value and [.] to the index.

**Table 2:** Comparisons of different LoRaWAN schemes.

	Standard LoRaWAN [51]	Na et al. [41]	Kim and Song [42]	Garcia et al. [43]	Garcia et al. [44]	Proposed
Scheme	Standard	XoR	Dual	Diameter	Radius	DO-SEO
Mutual Authentication	YES	YES	YES	YES	YES	YES
Secure Key Exchange	YES	YES	YES	YES	YES	YES
Perfect Forward Secrecy	NO	NO	NO	NO	NO	YES
End-to-End Security	NO	NO	NO	NO	NO	YES
Defense against a Replay Attack	NO	YES	NO	NO	NO	YES

**Table 3:** Burrows–Abadi–Needham (BAN)-logic notations.

Statement	Meaning
PbelievesX	*P* believes *X* and acts as if *X* were true.
PseesX	*P* receives *X* at present or in the past time.
PsaidX	*P* once said *X*, which means that *X* was sent to *P* at some point.
PcontrolsX	*P* has jurisdiction over *X*.
#(X)	*X* is fresh.
${\left\{X\right\}}_{K}$	*X* is encrypted with a secret *K*.
${\langle X\rangle }_{K}$	It means that *X* is combined with secret *K*. MIC can be expressed by this notation.
PKQ	*K* is a secret key only known to *P* and *Q*.
$\overset{K\;}{\to }P$	*K* is *P*’s public key.
PKQ	*K* is a secret only known to *P* and *Q*.

**Table 4:** Tiny DTLS-PSK 0.8.2 and Tiny DTLS-ECC 0.8.2 environments for evaluation.

Environment	Specific
CPU	Intel^®^ Core™i5-6300HQ 2.30 GHz (0.88 GHz)
RAM	8GB
Compiler	gcc (GCC) 6.4.0
OS	Windows 10 64bit (Cygwin32)
DTLS Library	TinyDTLS 0.8.2
Cipher Suite	TLS_ECDHE_ECDSA_WITH_AES_128_CCM_8
Cipher Suite	TLS_PSK_WITH_AES_128_CCM_8

**Table 5:** DTLS-PSK packet size and overheads.

Message	Size (Bytes)	Overhead (ms)
Client_Hello(1)	42	1
Hello_Verify_Request	19	-
Client Hello(2)	58	1
Server_Hello	38	-
Server_Hello_Done	0	-
Client_Key_Exchange	17	1
Finished(Client)	12	1
Finished(Server)_Verify	12	1
Total Overhead		5

**Table 6:** DTLS–PSK handshake message packet.

Message	Size (Bytes)
Client_Hello(1)	42
Hello_Verify_Request	19
Client_Hello(2)	58
Server_Hello, Server Hello Done	38
Client_Key_Exchange, Finished	29
Finished	12
Total Message Size	198

**Table 7:** DTLS-ECC packet size and overheads.

Message	Size (Bytes)	Overhead (ms)
Client_Hello(1)	72	1
Hello_Verify_Request	19	-
Client Hello(2)	88	1
Server_Hello	56	-
Certificate	97	1
Server_Key_Exchange	143	-
Certificate_Request	8	-
Server_Hello_Done	0	-
Client_Key_Exchange	66	31
Certificate_Verify	76	31
Finished(Client)	12	31
Finished(Server)	12	1 (for verification)
Total Overhead		97

**Table 8:** DTLS-ECC handshake message packet.

Message	Size (Bytes)
Client_Hello(1)	72
Hello_Verify_Request	19
Client_Hello(2)	88
Server_Hello, Certificate, Server_Key_Exchange, Certificate_Request, Server_Hello_Done	304
Certificate, Client_Key_Exchange, Certificate_Verify, Finished	251
Finished	12
Total Message Size	746

**Table 9:** Proposed protocol environment for evaluation.

Environment	Specific
CPU	Intel^®^ Core™i5-6300HQ 2.30 GHz (0.88 GHz)
RAM	8 GB
Compiler	Visual Studio 2017 32 bit
OS	Windows 10 64 bit

**Table 10:** Message size and overhead for the proposed protocol’s Default Option (DO).

Message	Size (Bytes)	Overhead (ms)
App_Auth_Req	37	1
APP_Auth_Res	53	9
App_Auth_ACK	4	1
Total Overhead		11
Total Message Size	94	

**Table 11:** Message size and overhead for the proposed protocol’s SEO.

Message	Size (Bytes)	Overhead (ms)
App_Auth_Req	69	3
APP_Auth_Res	53	12
App_Auth_ACK	4	1
Total Overhead		16
Total Message Size	126	

**Table 12:** System-based comparison for DTLS-PSK, DTLS-ECC and extended LoRaWAN protocol with DO and SEO features.

Protocol	Overhead (ms)	Overhead Impact w.r.t. DO	Overhead Impact w.r.t. SEO	Message Size (Bytes)	Message Size Impact w.r.t. DO	Message Size Impact w.r.t. SEO
DO	11	-	−31.25%	94	-	−25.39%
SEO	16	+31.25%	-	126	+25.39%	-
DTLS-PSK	5	−54.54%	−68.75%	198	+52.52%	+36.36%
DTLS-ECC	97	+88.65%	+83.50%	746	+87.39%	+83.10%

**Table 13:** Proposed protocol environment for evaluation over a mobile device.

Environment	Specific
Device Type	Mobile
Make	Lenovo
Chipset	Mediatek MT6753
CPU	Octa-core 1.3 GHz Cortex-A53
GPU	Mali-T720MP3
Compiler	Visual Studio 2017 (Android Kit)
OS	Android 5.1.1

**Table 14:** Overhead for the proposed protocol (DO and SEO) over a low-power device.

Message	DO (min) (ms)	DO (max) (ms)	SEO (min) (ms)	SEO (max) (ms)
*App_Auth_Req*	12	12	20	20
*APP_Auth_Res*	5	9	5	12
*App_Auth_ACK*	1	1	1	1
Total Overhead	18	22	26	33

**Table 15:** Parameter configurations.

Parameter	Value
N	10
B	11 Mbps
I	20 ms
F	64 Bytes
L2	45.35 ms
$T_{d}$	10–50 ms
K	1–10
τ	100 ms
$W_{d}$	20 ms
